# Cholesterol crystal depth in coronary atherosclerotic plaques: A novel index of plaque vulnerability using optical frequency domain imaging

**DOI:** 10.1371/journal.pone.0180303

**Published:** 2017-06-30

**Authors:** Masahiro Koide, Akiko Matsuo, Satoshi Shimoo, Kazuaki Takamatsu, Atsushi Kyodo, Yumika Tsuji, Kayoko Mera, Yoshinori Tsubakimoto, Koji Isodono, Tomohiko Sakatani, Keiji Inoue, Hiroshi Fujita

**Affiliations:** Department of Cardiovascular Medicine, Japanese Red Cross Society Kyoto Daini Hospital, Kyoto, Japan; Universitatsklinikum Freiburg, GERMANY

## Abstract

**Background:**

The involvement of cholesterol crystals (CCs) in plaque progression and destabilization of atherosclerotic plaques has been recently recognized. This study aimed to evaluate the association between the intraplaque localization of CCs and plaque vulnerability.

**Methods:**

We investigated 55 acute coronary syndrome (ACS) and 80 stable angina pectoris (stable AP) lesions using optical frequency domain imaging (OFDI) prior to percutaneous coronary intervention. The distance between CCs and the luminal surface of coronary plaques was defined as CC depth.

**Results:**

Although the incidence of CCs had similar frequencies in the ACS and stable AP groups (95% vs. 89%, p = 0.25), CC depth was significantly less in patients with ACS than in those with stable AP (median [25^th^ to 75^th^ percentile]: 68 μm [58 to 92 μm] vs. 152 μm [115 to 218 μm]; p < 0.001). The incidences of plaque rupture, thrombus, lipid-rich plaques, and thin-cap fibroatheroma were significantly greater in patients with ACS than in those with stable AP (62% vs. 18%, p < 0.001; 67% vs. 16%, p < 0.001; 84% vs. 57%, p < 0.01; and 56% vs. 19%, p < 0.001, respectively).

**Conclusion:**

OFDI analysis revealed that CCs were found in the more superficial layers within the coronary atherosclerotic plaques in patients with ACS than in those with stable AP, suggesting that CC depth is associated with plaque vulnerability. CC depth, a novel OFDI-derived parameter, could be potentially used as an alternative means of evaluating plaque vulnerability in coronary arteries.

## Introduction

Despite the significant development of pharmacological therapeutics, cardiovascular disease remains the major cause of death in developed countries [1]. It has been recognized that acute coronary syndrome is induced by the erosion and rupture of vulnerable atherosclerotic plaques; thus, because the early detection of vulnerable, rupture-prone atherosclerotic plaques is expected to lead to improved cardiovascular event prevention, vigorous research is being conducted using various imaging modalities, including intravascular ultrasound (IVUS) [2], near-infrared spectroscopy [3], computed tomography angiography [4], magnetic resonance imaging [5], positron emission tomography [6], and optical coherence tomography (OCT) [7].

OCT is a novel intravascular imaging modality that uses the reflection of near-infrared light to create images. OCT achieves high-resolution images between 10 to 20 μm, which is 10 times higher than the resolution of IVUS. The most representative OCT findings indicating vulnerable plaques that may lead to acute coronary syndrome (ACS) are lipid-rich plaques and thin-cap fibroatheroma (TCFA), which are confirmed by histopathological analysis [8,9]. Furthermore, macrophage accumulation [10], microvessels within atherosclerotic plaques [11], and adventitial vasa vasorum [12] are known to be other indicators of vulnerable plaques. However, previous prospective trials evaluating plaque vulnerability by intravascular imaging demonstrated a low predictive value for subsequent lesion progression and future cardiovascular events [11,13]. Therefore, a novel index to efficiently detect for vulnerable plaques is needed.

An increased high local concentration of esterified cholesterol in atherosclerotic plaques leads to the crystallization of cholesterol. Therefore, cholesterol crystals (CCs) are familiar hallmarks of atherosclerotic lesions and are considered to be passive elements of necrotic cores [14]. However, recent research has indicated that the phagocytosis of CCs by macrophages actively induces plaque progression and destabilization through intraplaque inflammation via the stimulation of the nucleotide-binding domain and leucine-rich repeat-containing protein 3 (NLRP3) pathway [15]. Furthermore, in clinical settings, the presence of CCs in coronary stenotic lesions has been associated with plaque vulnerability by OCT analysis [16,17,18].

Optical frequency domain imaging (OFDI) is a second-generation OCT with a higher frame rate and pullback speed [19]. We hypothesized that intraplaque localization of CCs is associated with plaque vulnerability, and thus, we estimated the CC localization using OFDI.

## Materials and methods

### Study population

In this study, we evaluated 135 coronary culprit lesions of consecutive patients who had undergone successful OFDI before percutaneous coronary intervention (PCI). Patients with the following medical conditions were excluded: left main coronary artery disease, previous intervention in a culprit vessel, cardiogenic shock, and undergoing rotational atherectomy before OFDI. The 135 enrolled lesions were divided into two groups according to the patient’s clinical course: the ACS group (n = 55), which included ST-elevation myocardial infarction (STEMI), non-STEMI (NSTEMI), and unstable angina, and the stable angina pectoris (stable AP) group (n = 80). STEMI and NSTEMI were defined according to recent guidelines [20]. Unstable angina was defined as new-onset angina, a progressive crescendo pattern of angina, or angina at rest within 2 weeks. The culprit lesions of ACS were confirmed by ECG, coronary angiographic, and echocardiographic findings. This study was approved by the review board of the Japanese Red Cross Society Kyoto Daini Hospital (Kyoto, Japan), and all patients provided written, informed consent.

### Angiographic analysis

Coronary angiography was performed with a 6-Fr guiding catheter through the radial or femoral artery after intracoronary administration of 100 μg of nitroglycerin. Angiographic analysis was performed using a quantitative coronary analysis (QCA) program (Heart II^™^, Gadelius Medical Corporation, Tokyo, Japan).

### OFDI analysis

An OFDI imaging catheter (FastView^™^, Terumo Corporation, Tokyo, Japan) was advanced with a 0.014-inch guide wire, and the imaging core was placed at the distal site of the culprit lesion. Lunawave^™^ (Terumo corporation, Tokyo, Japan) was used as an imaging console for the OFDI system. OFDI was generally performed without dilation by a balloon catheter; however, if the OFDI catheter could not pass through the lesion because of severe stenosis, the lesion was dilated with a small-sized balloon. Patients without spontaneous recanalization in the ACS group were subject to thrombus aspiration before OFDI. The plaque morphology of culprit lesions was investigated using OFDI. Culprit lesions were defined as the area in which stents were implanted during PCI. OFDI analysis was conducted offline at each cross section by two independent investigators (M.K. and A.M.) who were blinded to each patient’s clinical course.

**Fig 1:**
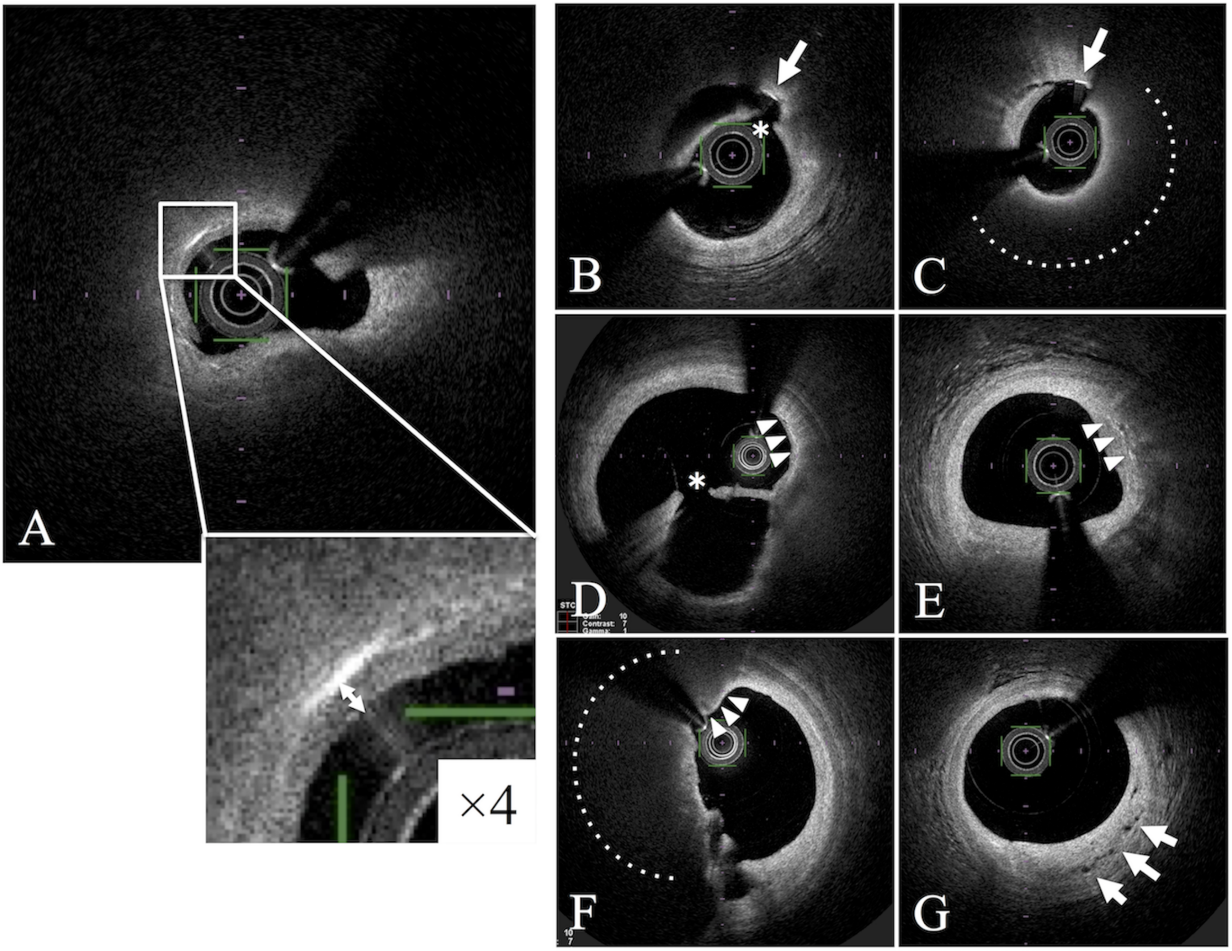
Cholesterol crystals (CCs), CC depth and other OFDI-derived vulnerable features. A. A representative OFDI image visualizing cholesterol crystals within coronary atherosclerotic plaques in patients with ACS. A cholesterol crystal was defined as a thin, linear region of high signal intensity within the lipid plaque, without backscattering (white box). The distance between the shallowest cholesterol crystals (CCs) and the luminal surface in the culprit lesion was defined as CC depth (white arrow). B-C. Exposed cholesterol crystals with uncovered fibrous caps were excluded from evaluation (white arrow). D-E. Macrophage accumulations (white arrowheads). F. TCFA is indicated by white arrowheads. G. Microchannels are indicated by white arrows. Dotted lines indicate lipid core. * indicates ruptured fibrous cap.

A CC was defined as a thin, linear region of high-signal intensity within the lipid plaque without backscattering [21]. The distance between the luminal surface and the shallowest CCs in the culprit lesion were defined as CC depth (Fig 1). Protruding or exposed CCs on the outside of the plaque were not measured; only CCs within the lipid plaque covered with a fibrous cap were evaluated. OFDI analysis revealed the presence of plaque rupture, thrombus, lipid-rich plaque, TCFA, macrophage accumulation, microvessels, and CCs in the plaque as well as CC depth. Lipid-rich plaques were defined as lesions with a lipid arc of more than 180° [21]. TCFA was defined as a fibrous cap thickness of less than 65 μm [21]. Fibrous cap thickness was defined as the minimum thickness of a signal-rich layer from the coronary artery lumen to the inner border of the underlying lipid in the culprit lesion [21]. The OFDI analysis of the fibrous cap thickness was exceptionally performed using the sepia scale measurement because it was easier to measure and visualize the fibrous cap than that using the gray scale. Macrophage accumulation was defined as signal-rich, distinct, or confluent punctate regions exceeding the intensity of the background speckle noise, between the bottom of the cap and the top of the necrotic core [21]. Microvessels were defined as vessels within the intima, which were well-delineated regions or voids with low backscattering [21]. Regarding the distinction between CC and macrophage accumulation in bright structures in OFDI images, CCs were defined as a linear region without backscattering, whereas aggregates in a punctate region with backscattering were defined as macrophage accumulations.

### Statistical analysis

Statistical analysis was performed with JMP^™^ version 9 for Windows (SAS institute, Cary, NC, USA). Categorical data are expressed as n (%) and compared using the chi-square test. Continuous variables with normal distributions are expressed as the mean ± standard deviation (SD) or the median (25^th^ to 75^th^ percentile) and compared using the Student’s t-test. Pearson’s correlation coefficient (r) was used to evaluate correlations between CC depth and fibrous cap thickness. Receiver-operating characteristic (ROC) curve analysis was performed to determine the optical threshold for CC depth to predict ACS plaque. The cut-off point was defined as the greatest sum of the sensitivity and specificity estimates. A p value < 0.05 was considered statistically significant.

## Results

Baseline patient characteristics are summarized in Table 1. No significant differences were observed between groups for baseline characteristics, such as comorbidities related to glucose metabolism, hyperlipidemia, lipid profile, and statin use, expect for renal function, in both groups. Lesion characteristics are shown in Table 2. QCA and OFDI analysis revealed that lesion stenosis before the PCI procedure was more severe in the ACS group than in the stable AP group (Table 2).

**Table 1:** Patient baseline characteristics.

	Stable AP (n = 80)	ACS (n = 55)	p
Age	68.6 ± 10.6	70.3 ± 9.7	0.32
Male (%)	67/80 (84%)	44/55 (80%)	0.58
Coronary risk factor			
Hypertension	62/80 (78%)	38/55 (69%)	0.27
Diabetes mellitus	35/80 (44%)	21/55 (38%)	0.52
Hyperlipidemia	49/80 (61%)	37/55 (67%)	0.47
Smoking	49/80 (61%)	33/55 (60%)	0.99
Family history	17/80 (21%)	16/55 (29%)	0.27
LVEF (%)	62.6 ± 11.9	60.3 ± 10.4	0.26
eGFR (ml/min/1.73 m^2^)	58.3 ± 21.0	68.0 ± 16.1	< 0.01*
CKD	31/80 (39%)	11/55(20%)	0.02*
HbA1c	6.5 ± 1.3	6.6 ± 1.1	0.63
LDL-C	111.8 ± 32.2	118.5 ± 28.7	0.21
HDL-C	52.2 ± 13.8	51.0 ± 14.5	0.63
TG	148.2 ± 91.7	132.8 ± 81.7	0.31
Statin use	44/80 (55%)	36/55 (65%)	0.06

Values are represented as mean ± SD or n (%); LVEF: left ventricular ejection fraction; eGFR: estimated glomerular filtration rate; CKD: chronic kidney disease; HbA1c: hemoglobin A1c; LDL-C: low-density lipoprotein cholesterol; HDL-C: high-density lipoprotein cholesterol; TG: triglyceride;

* denotes statistical significance (p < 0.05).

**Table 2:** Lesion characteristics.

	Stable AP (n = 80)	ACS (n = 55)	p
Lesion			0.29
LAD	48/80 (60%)	29/55 (53%)	
LCx	14/80 (18%)	7/55 (13%)	
RCA	18/80 (23%)	19/55 (35%)	
Type B2/C	49/80 (61%)	40/55 (73%)	0.17
QCA analysis			
Pre % stenosis	65.7 ± 13.1	83.3 ± 15.4	< 0.001*
Reference diameter	2.8 ± 0.5	3.0 ± 0.6	0.11
Pre MLD	1.0 ± 0.4	0.5 ± 0.5	< 0.01*
Lesion length	21.4 ± 11.2	24.1 ± 13.8	0.23
OFDI analysis			
Distal RVA	10.1 ± 4.2	11.9 ± 4.9	0.06
MLA	1.5 ± 0.7	1.3 ± 0.8	0.04*
MLD	1.3 ± 0.3	1.1 ± 0.4	0.03*
Lesion length	23.1 ± 11.1	26.3 ± 12.5	0.1

Values are represented as mean ± SD or n (%); LAD: left anterior descending coronary artery; LCx: left circumflex artery; RCA: right coronary artery; QCA: quantitative coronary angiogram analysis; Pre % stenosis: percent of stenosis before PCI; MLD: minimal lumen diameter; RVA: reference vessel area; MLA: minimal lumen area;

* denotes statistical significance (p < 0.05)

**Fig 2:**
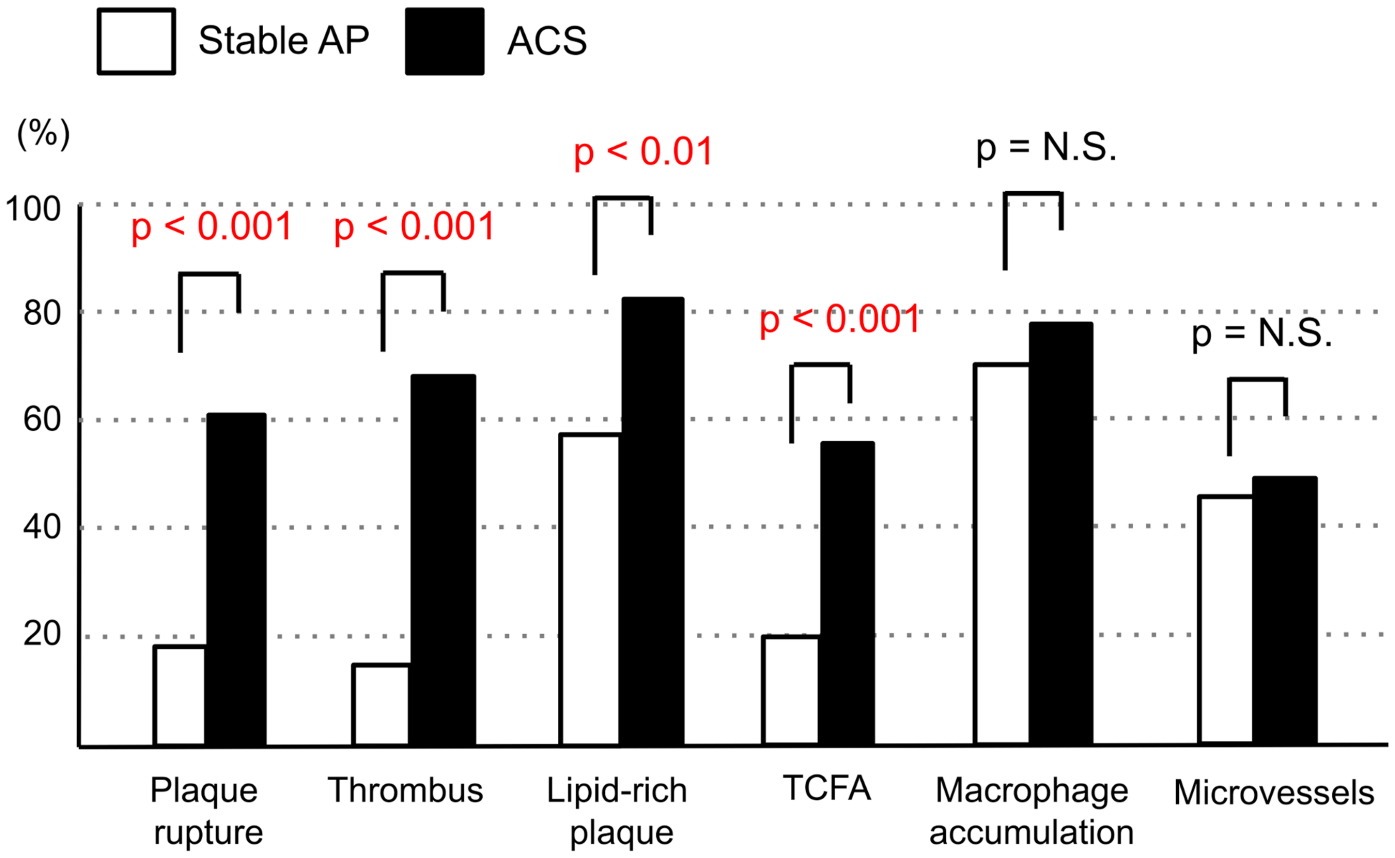
Results of plaque analysis by OFDI in the stable AP and ACS groups. TCFA: thin-cap fibroatheroma. The y-axis indicates the % of cases.

The results of plaque analysis by OFDI are shown in Fig 2. The culprit lesions in the ACS group had higher incidences of plaque rupture (62% vs. 18%, p < 0.001), thrombus (67% vs. 16%, p < 0.001), lipid-rich plaques (84% vs. 57%, p < 0.001), and TCFA (56% vs. 19%, p < 0.001) than those in the stable AP group (Fig 2). Conversely, there was no significant difference in the presence of macrophage accumulation (78% vs. 71%, p = 0.37) and microvessels (51% vs. 44%, p = 0.36) between the groups (Fig 2).

**Fig 3:**
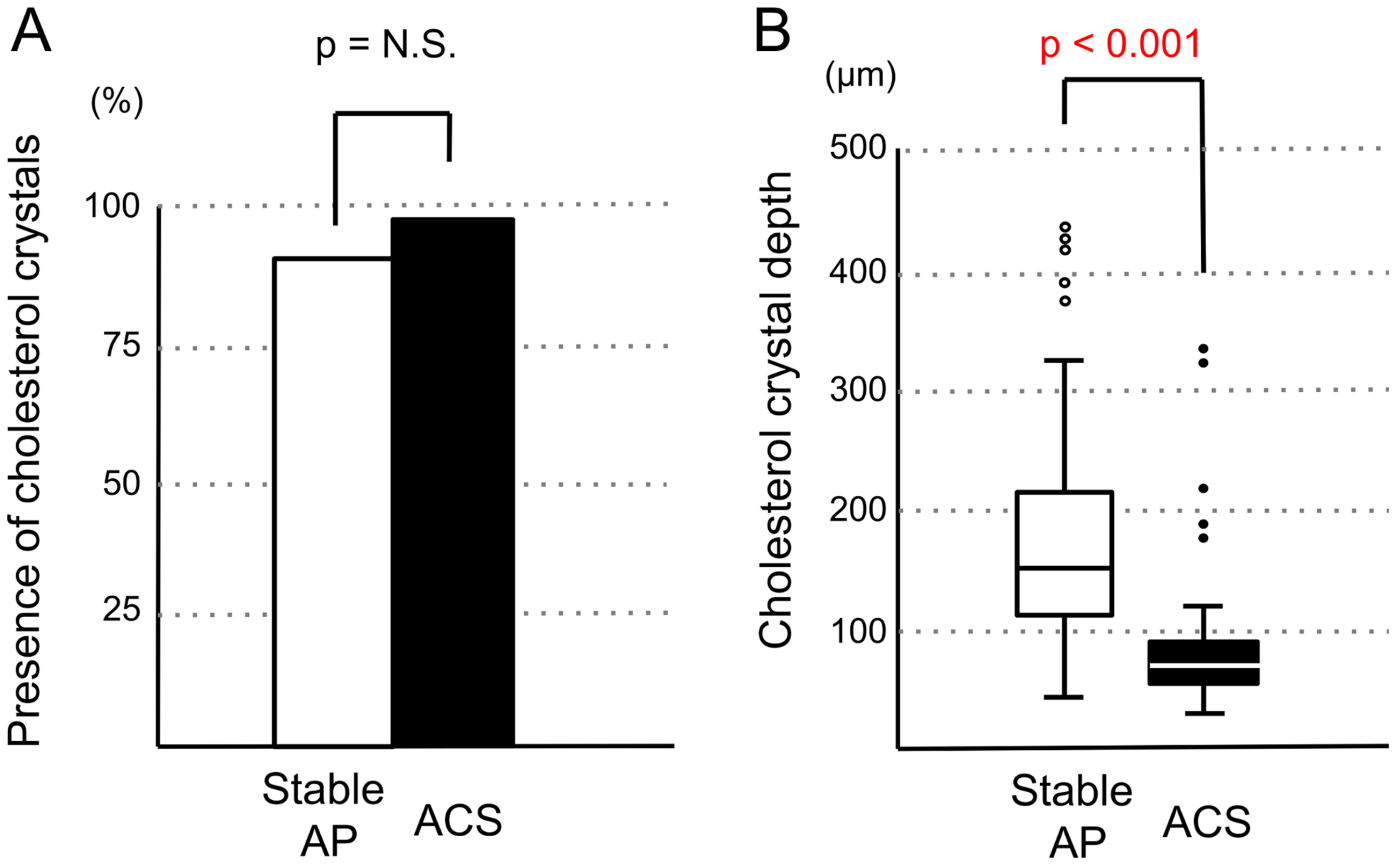
Presence of cholesterol crystals (A) and CC depth (B) in the stable AP and ACS groups. The presence of cholesterol crystals and CC depth in the culprit lesion undergoing PCI were compared between the ACS and stable AP groups. The y-axis in Fig 3A indicates the % of cases.

**Fig 4:**
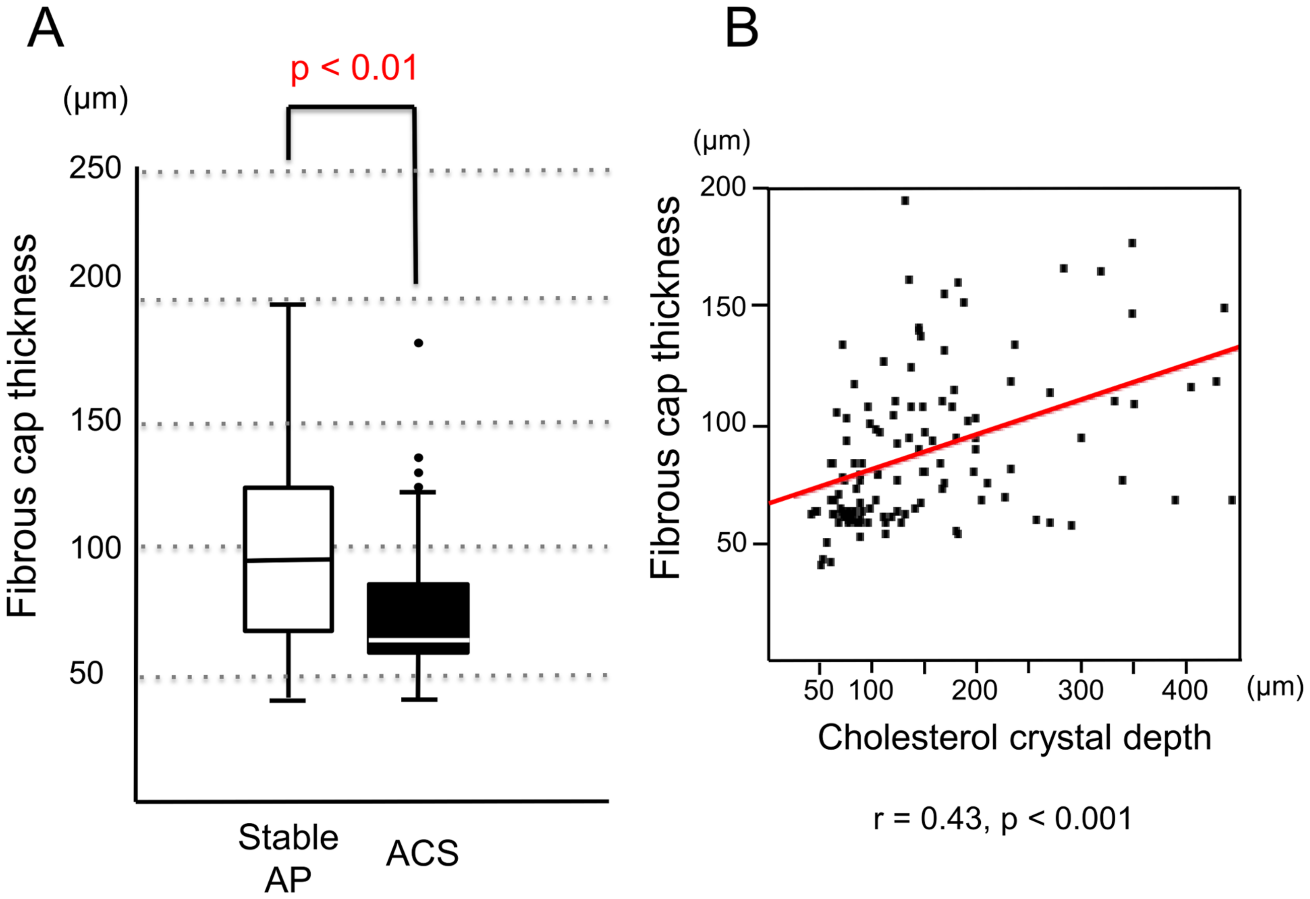
**(A) Comparison of fibrous cap thickness between the stable AP and ACS groups. (B) Correlation between cholesterol crystal presence and fibrous cap thickness.** The minimum fibrous cap thickness in the culprit lesion undergoing PCI was compared between the Stable AP and ACS groups.

Although the presence of CCs in the culprit lesions had similar frequencies in the ACS and stable AP groups (95% vs. 89%, p = 0.25), CC depth was significantly less in patients with ACS than in those with stable AP (median [25^th^ to 75^th^ percentile]: 68 μm [58 to 92 μm] vs. 152 μm [115 to 218 μm], p < 0.001; Fig 3). Fibrous cap thickness was significantly less in the ACS group than in the stable AP group (66 μm [62 to 85 μm] vs. 96 μm [69 to 125 μm], p < 0.01; Fig 4a). However, the correlation between CC depth and fibrous cap thickness was significant but weak (r = 0.43, p < 0.001; Fig 4b).

**Fig 5:**
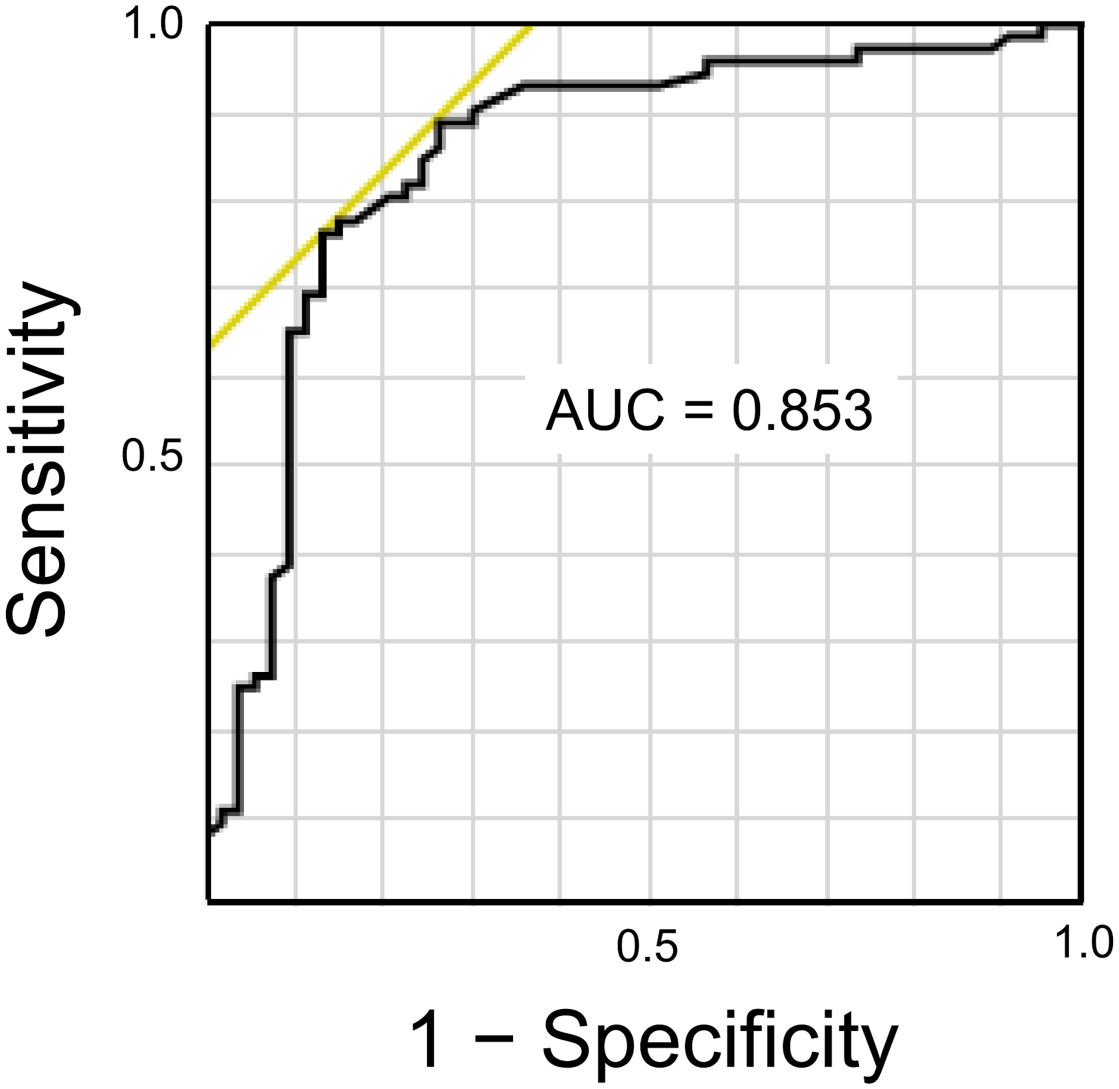
ROC curve analysis calculating the cut-off value of CC depth to identify the ACS plaques. The area under the curve (AUC) was 0.853. A cut-off value of CC depth of 115 μm had a sensitivity of 76% and specificity of 87% in predicting the ACS plaques.

The ROC curve to calculate the cut-off value of CC depth to predict the culprit lesion plaques in patients with ACS is shown in Fig 5. A cut-off value of CC depth of 115 μm had a sensitivity of 76% and specificity of 87% to predict the presence of ACS plaques.

**Fig 6:**
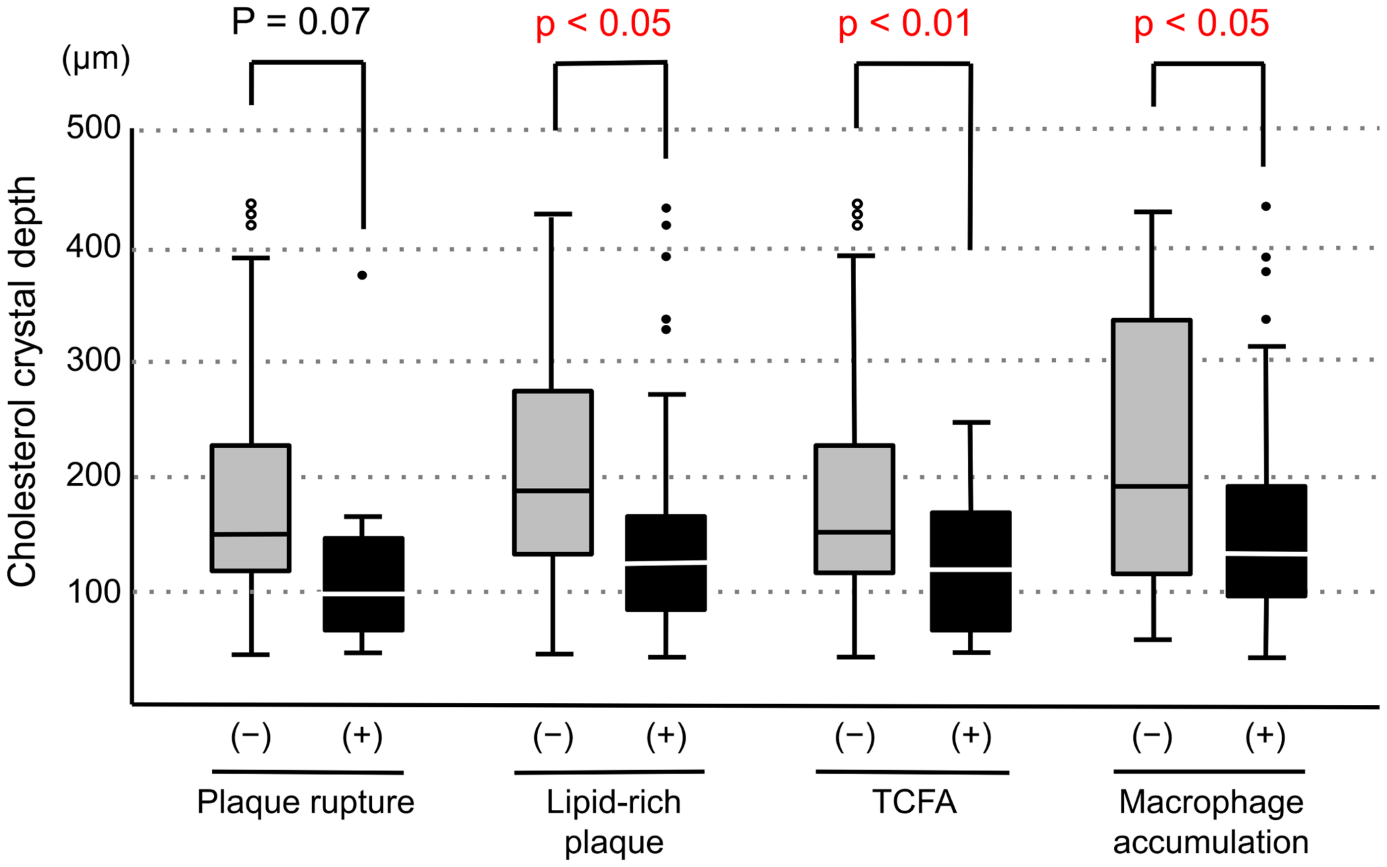
Comparison of CC depth according to the presence or absence of plaque rupture, lipid-rich plaque, TCFA and macrophage accumulation in the lesion without thrombus. CC depth: Cholesterol crystal depth, TCFA: thin-cap fibroatheroma.

**Fig 7:**
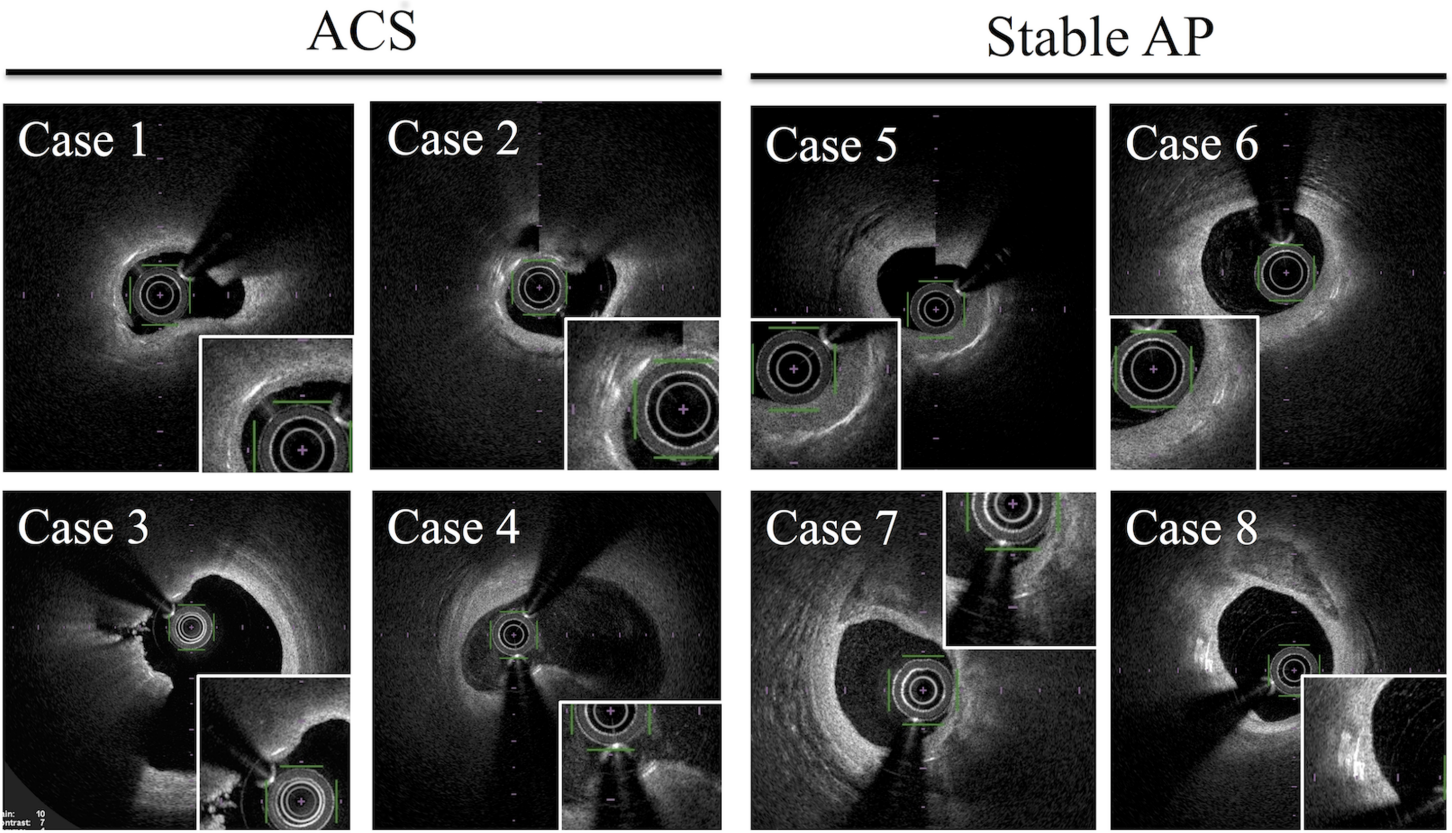
Representative OFDI findings in patients with ACS and stable AP.

In 85 lesions in which thrombus were not detected by OFDI, we compared CC depth according to the presence or absence of plaque rupture, lipid-rich plaque, TCFA, and macrophage accumulation. CC depth was significantly less in culprit lesions with the presence of lipid-rich plaque, TCFA, and macrophage accumulation (Fig 6). Representative OFDI findings in patients with ACS and stable AP were shown in Fig 7.

## Discussion

The main finding of this study was that CCs were more frequently found in the more superficial layers of coronary culprit lesions in patients with ACS undergoing PCI than in those with stable AP, while the incidence of CCs was similar between the two groups. To the best of our knowledge, this is the first report to evaluate the relation between the intraplaque localization of CCs and plaque vulnerability. It has been reported that the defining characteristics of vulnerable plaques, as determined by OCT and OFDI, are the presence of lipid-rich plaques, TCFA, macrophage accumulation, microvessels, and others. In this study, we have proposed a novel index of plaque vulnerability, the “CC depth,” which is the depth of CCs in atherosclerotic plaques as identified by OFDI.

In previous reports, which employed OCT, CCs were found in 39%–40% of culprit lesions or vessels in patients with stable angina undergoing PCI [17,22] and in 39% of culprit lesions in patients with ACS [16]. These lesions containing CCs exhibited OCT-derived plaque vulnerability features, suggesting the association between the incidence of CCs and plaque vulnerability [16,17]. However, our data obtained by OFDI revealed that the incidence of CCs in the culprit lesion had similar high frequencies among the ACS and stable AP groups (Fig 3). Although the reasons for this are unknown, it may be possible that minuscule CCs are detectable by OFDI because they appear bright due to the higher power of the OFDI system than that of the OCT system.

The plaque contents, including the lipid core and crystals, often protruded and were exposed to the vessel lumen in patients with ACS; therefore, we excluded the protruded crystals from the evaluation and made efforts to measure only crystals within the lipid plaque covered with a fibrous cap. However, it might be difficult to completely discriminate between protruding plaque contents and original plaque contents. To address these methodological concerns, we compared the CC depth according to the presence or absence of plaque rupture, lipid-rich plaque, TCFA and macrophage accumulation only in the lesions in which thrombus and plaque protrusion were not observed. Therefore, CC depth was significantly less in culprit lesions in which we found the OCT-derived plaque vulnerability features (Fig 6).

Nishimura et al demonstrated that the presence of CCs in coronary plaques are associated with clinical metabolic disorders and OCT-derived vulnerable morphological features [18]. They also showed that CCs in the plaques with thrombus located in the more superficial layer of plaques than those in the plaques without thrombus, regardless of whether the patients had ACS or stable AP. However, they did not evaluate the relationship between CC localization and other OCT-derived vulnerable features, and they did not describe the significance of their results. Furthermore, they did not describe their methods for assessing protruded or exposed CCs around ruptured plaques, although exposed CCs with uncovered fibrous caps were frequently observed around plaques with thrombus. Our study excludes exposed CCs with uncovered fibrous caps from the evaluation and provides the first demonstration of the significance of CC depth for plaque vulnerability.

In this study, CCs were found in the more superficial plaque layers of patients with ACS than in those with stable AP (Fig 3). Furthermore, fibrous cap thickness was significantly less in the culprit lesions of patients with ACS than in those of patients with stable AP. In OFDI, the visualization of CCs was limited to only the superficial layer of the lipid core because of high attenuation owing to the lipid component. Thus, there may be an association between the most superficial CCs and thinnest fibrous cap. In fact, a significant but weak correlation was observed between these two variables (Fig 4b). However, there were not many cases where the most superficial crystals were located directly below the thinnest fibrous caps. Therefore, we considered that CC depth is not just dependent on the thickness of the thinnest fibrous cap.

Previous studies have shown that intraplaque macrophages phagocytose CCs, which leads to the production of interleukin 1 beta (IL-1 beta) through the assembly of the NLRP3 inflammasome [15]. Additionally, macrophages are activated by IL-1 beta and overproduce matrix metalloproteinases, which are specific enzymes with interstitial collagenase activity, which makes fibrous caps thinner through the degradation of the interstitial type I collagen-composed cap [23]. Therefore, CCs that exist near the fibrous cap could be implicated in plaque destabilization by advancing the thinning of the fibrous cap.

Other previous studies have demonstrated that cholesterol expands in volume during crystallization from the liquid to the solid state and forms sharp-tipped edges [24,25]. Autopsy findings of patients who died of myocardial infarction have shown that CCs perforate the intimal surface overlying ruptured plaques not only in the culprit artery but also in other coronary arteries of the same heart [26]. In that paper, the researchers hypothesized that cholesterol expands in the limited space of the necrotic core as it crystallizes, generating sharp-tipped crystals that eventually pierce the fibrous cap, leading to plaque erosion and rupture [25]. The more superficial distribution of crystals in plaques makes it easier to perforate the fibrous cap. Therefore, CCs distributed in shallower layers may be associated with plaque vulnerability through mechanical injury to the fibrous cap.

Several limitations of the present study should be mentioned. First, this was a retrospective study conducted at a single institution, and its sample size was relatively small; therefore, potential selection bias could not be avoided. Second, because we distinguished between ACS and stable AP on the basis of the clinical course, the ACS group also contained patients with myocardial infarction secondary to fixed atherosclerosis and supply-demand ischemia imbalance, as well as plaque rupture with thrombosis. However, these fixed atherosclerotic lesions might not be histologically identified as vulnerable plaques. Third, it is still unclear whether CC depth can offer any additional information and values over minimum fibrous cap thickness and presence of macrophages which have been already established vulnerability criteria. In order to evaluate the value of this novel index, the CC depth, a prospective study is necessary to investigating whether the depth of CCs within the intermediate stenotic lesion has an impact on future clinical events.

## Conclusion

By OFDI analysis, CCs were more frequently found in the more superficial layers of coronary atherosclerotic plaques in patients with ACS than in those with stable AP. This suggests that CC depth is associated with plaque vulnerability. The OFDI-derived novel parameter, CC depth, could potentially be used as an alternative measurement to probe the vulnerability of plaques in coronary arteries.
